# Transcriptional comparison of the filamentous fungus *Neurospora crassa* growing on three major monosaccharides D-glucose, D-xylose and L-arabinose

**DOI:** 10.1186/1754-6834-7-31

**Published:** 2014-02-28

**Authors:** Jingen Li, Liangcai Lin, Huiyan Li, Chaoguang Tian, Yanhe Ma

**Affiliations:** 1 Key Laboratory of Systems Microbial Biotechnology, Tianjin Institute of Industrial Biotechnology, Chinese Academy of Sciences, Tianjin 300308, China; 2 Institute of Microbiology, Chinese Academy of Sciences, Beijing 100101, China; 3 University of Chinese Academy of Sciences, Beijing 100049, China

**Keywords:** *Neurospora crassa*, L-arabinose, RNA-seq, GLT-1, XAT-1, XYT-1, HCR-1

## Abstract

**Background:**

D-glucose, D-xylose and L-arabinose are the three major monosaccharides in plant cell walls. Complete utilization of all three sugars is still a bottleneck for second-generation cellulolytic bioethanol production, especially for L-arabinose. However, little is known about gene expression profiles during L-arabinose utilization in fungi and a comparison of the genome-wide fungal response to these three major monosaccharides has not yet been reported.

**Results:**

Using next-generation sequencing technology, we have analyzed the transcriptome of *N. crassa* grown on L-arabinose versus D-xylose, with D-glucose as the reference. We found that the gene expression profiles on L-arabinose were dramatically different from those on D-xylose. It appears that L-arabinose can rewire the fungal cell metabolic pathway widely and provoke the expression of many kinds of sugar transporters, hemicellulase genes and transcription factors. In contrast, many fewer genes, mainly related to the pentose metabolic pathway, were upregulated on D-xylose. The rewired metabolic response to L-arabinose was significantly different and wider than that under no carbon conditions, although the carbon starvation response was initiated on L-arabinose. Three novel sugar transporters were identified and characterized for their substrates here, including one glucose transporter GLT-1 (NCU01633) and two novel pentose transporters, XAT-1 (NCU01132), XYT-1 (NCU05627). One transcription factor associated with the regulation of hemicellulase genes, HCR-1 (NCU05064) was also characterized in the present study.

**Conclusions:**

We conducted the first transcriptome analysis of *Neurospora crassa* grown on L-arabinose and performed a comparative analysis with cells grown on D-xylose and D-glucose, which deepens the understanding of the utilization of L-arabinose and D-xylose in filamentous fungi. The dataset generated by this research will be useful for mining target genes for D-xylose and L-arabinose utilization engineering and the novel sugar transportes identified are good targets for pentose untilization and biofuels production. Moreover, hemicellulase production by fungi could be improved by modifying the hemicellulase regulator discovered here.

## Background

The pentose sugars L-arabinose and D-xylose, along with D-glucose, are the three most abundant components of plant biomass. Consumption of D-xylose and L-arabinose and co-fermentation with D-glucose is essential for complete utilization of carbon during biomass refining, which is critical for the economic production of cellulosic fuels and chemicals [1].

Compared with L-arabinose, much research has focused on both the enzymes of the D-xylose utilization pathway and the genome-wide response to D-xylose in yeast and filamentous fungi. In fungi, D-xylose is converted to D-xylitol, then to D-xylulose and finally phosphorylated to D-xylulose-5-phosphate, which goes into the pentose phosphate pathway, via the enzymes D-xylose reductase (XR), xylitol dehydrogenase (XDH) and xylulokinase (XKS), respectively. The characteristics of XR and XDH from different species have been analyzed and significant improvement of xylose-fermentating yeast has been achieved by engineering of XR and XDH [2,3]. Many fungi can use D-xylose as a sole carbon source, and 14 xylose-fermenting fungal genomes have been sequenced and compared [4]. Genome-wide analysis of the response to D-xylose has also been reported in multiple fungi, such as *Aspergillus niger*, *Neurospora crassa*, *Scheffersomyces stipitis* and engineered *Saccharomyces cerevisiae*[5-8]. The sequenced genomes and transcriptome analysis open a great repository of target gene mining for xylose-fermenting yeast engineering, including D-xylose transporters. Several pentose-specific transporters have been cloned and characterized to date, such as *Stp2*, *Gxfl*, *An25* and *Xyp29*[9-11]. In *A. niger*, D-xylose can serve as an inducer for cellulase and hemicellulase genes [12,13], while in *Trichoderma reesei*, D-xylose is able to induce or repress the expression of hemicellulases dependent on concentration [14]. D-xylose utilization and hemicellulase expression in both organisms are regulated by XlnR/XYR1 [15,16]. In *N. crassa*, xylanase expression and D-xylose utilization are also regulated by XYR-1, an ortholog of XlnR/XYR1. Neither growth nor xylanase activity was detected in a *xyr-1* deletion strain when it was exposed to D-xylose or xylan [8].

Compared to D-xylose metabolism, L-arabinose needs only two more enzymes, L-arabitol dehydrogenase and L-xylulose reductase, to form xylitol before its confluence into the pentose phosphate pathway [17]. Many filamentous fungi can utilize both pentoses as a sole carbon source, because of the significant overlap between two sugars’ catabolic pathways. Nevertheless, differences in the regulation of their utilization have been reported in *A. niger*, the L-Arabinose was regulated by AraR whereas the D-xylose was regulated by XlnR, although some overlaps occurs between the AraR and XlnR regulation network [18,19]*.* Therefore, we set out to test whether the genome-wide response of microbes to L-arabinose at the mRNA level is similar or different to that of D-xylose. This knowledge will be really useful for subsequent industrial microbial engineering, in both yeast and filamentous fungi, for the production of chemicals and fuels from pentose sugars. To date in fungi, there has been no transcriptome analysis on L-arabinose. We performed transcriptome analysis of fungi grown on L-arabinose, D-xylose or D-glucose by RNA sequencing using *N. crassa,* as a model filamentous fungus, has been used extensively for genetic, biochemical, and molecular studies [20] and has the capability of using either D-xylose or L-arabinose as sole carbon sources. The enzymes involved in the pentose catabolic pathway of *N. crassa* have been cloned and characterized for functionality [21-24]. With great tools and resources, *N. crassa* has become a model for studying the mechanism of the lignocellulosic degradation, cellulase expression regulation and sugar transport [1].

This is the first transcriptome analysis of filamentous fungi growing on L-arabinose, and the transcriptional comparison sheds much light on the molecular basis of genome-wide microbial responses to two pentose sugars. Based on RNA-seq data of the present study, dramatic differences of transcriptional profiles under L-arabinose and D-xylose were found. Many more genes showed differential expression on L-arabinose, including genes with no obvious function related to pentose metabolism. And over half of sugar transporters of the genome were upregulated, and over 20 transcription factors were induced by L-arabinose significantly. Finally, the dataset generated by this research will be valuable for target gene mining for D-xylose or L-arabinose utilization for subsequent microbe engineering, particularly the novel pentose transporters (GLT-1, XAT-1 and XYT-1) and the transcription factors (hemicellulase regulator -1 (HCR-1)) identified.

## Results

### The growth of *Neurospora cra*ssa on D-glucose, D-xylose and L-arabinose

**Figure 1:**
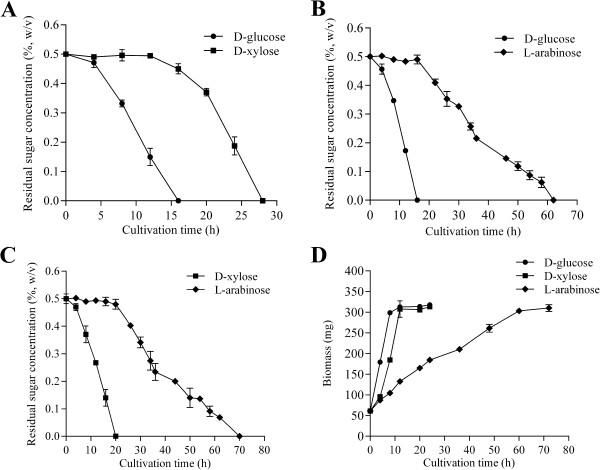
**Sugar consumption of *Neurospora crassa *mycelia precultivated on Vogel’s minimal medium with 2% D-glucose for 16 h then transferred to minimal medium with the following sugar mixtures (all 0.5%). (A)** D-glucose and D-xylose, **(B)** D-glucose and L-arabinose, **(C)** D-xylose and L-arabinose. **(D)** The grow rate of wild-type (WT) on each of three monosacchrides. The mycelia, which were pre-cultured on D-glucose for 16 h were washed, transferred to minimal medium supplemented with 0.5% D-glucose, 0.5% D-xylose or 0.5% L-arabinose, respectively, and the dry weight of the mycelia were determined as growth biomass at different intervals. The values are the means of two independent experiments.

To determine the utilization efficiency of D-glucose, D-xylose and L-arabinose in *N. crassa*, mycelia pre-cultured on D-glucose for 16 h were transferred to mixed sugar and the speed and pattern of sugar utilization were measured (See Methods and Figure 1). Figure 1A-C shows that when D-glucose was at a low level concentration (about 0.16%), the cell started to use D-xylose and D-glucose simultaneously. In contrast, L-arabinose consumption started only after the complete depletion of D-glucose and D-xylose (Figure 1). As for most microbes, the sequence of sugar utilization is D-glucose > D-xylose > L-arabinose. For 0.5% initial sugar, it took about 16 h to use up D-glucose, and 20 h and 50 h were required to deplete the D-xylose and L-arabinose (respectively) in the same conditions. The growth rate of the wild-type (WT) cell on each of three monosaccharides reflected their sugar consumption rate and preference (Figure 1D).

**Figure 2:**
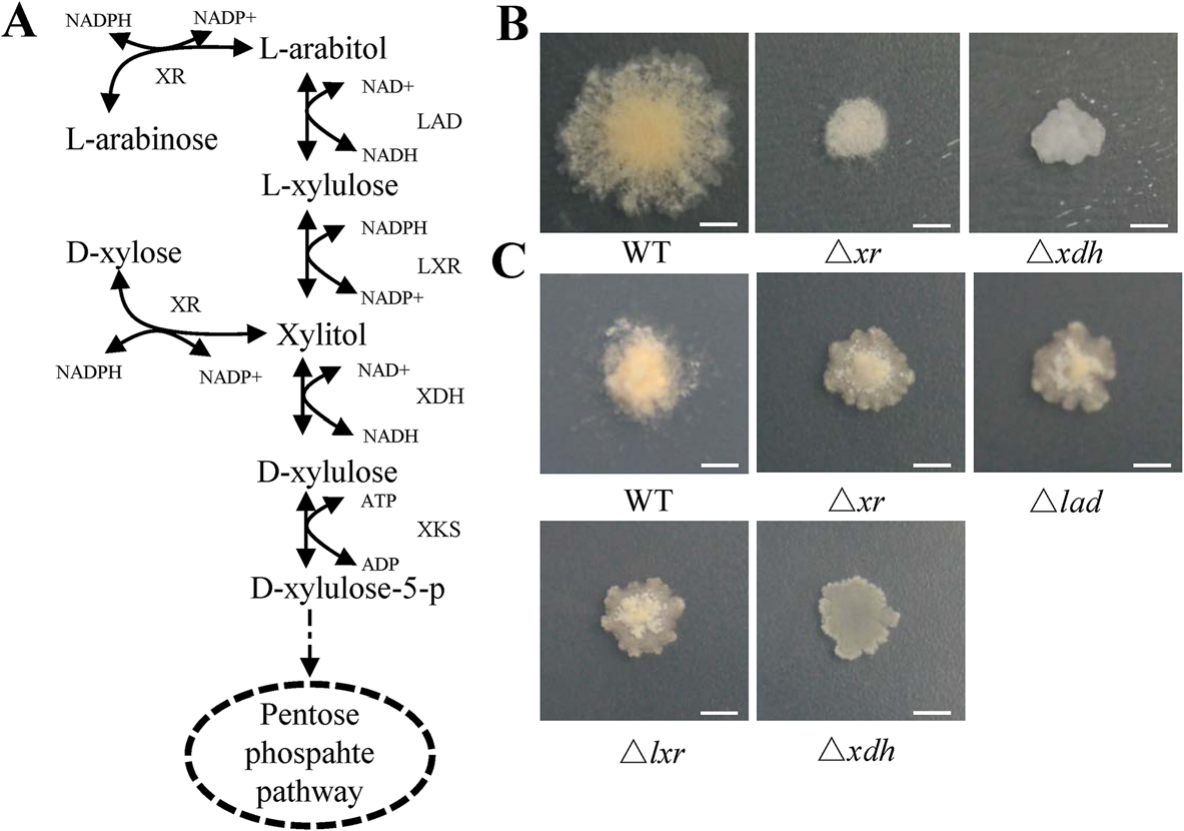
**The genes involved in pentose metabolism of *N. crassa. *(A)** General scheme for pentose catabolic pathway in fungi. **(B)** Growth of strains carrying a deletion of genes involved in the xylose catabolic pathway on BDES plate [26] plus 2% D-xylose for 3 days. **(C)** Growth of strains carrying a deletion of the gene involved in the L-arabinose catabolic pathway on BDES plate plus 2% L-arabinose for 5 days. XR, D-xylose reductase, NCU08384: XDH, xylitol dehydrogenase, NCU00891; LAD, L-arabitol dehydrogenase, NCU00643; LXR, L-xylulose reductase, NCU09041; NADH, nicotinamide adenine dinucleotide, reduced; NADPH, nicotinamide adenine dinucleotide phosphate, reduced; WT, wild-type. The length of the scale is about 0.5 cm.

Taking advantage of whole genome gene deletion strains, we tested the phenotypes of predicted knock-outs in the basic pentose metabolic pathway genes when cells were grown on D-xylose or L-arabinose. Genes tested included D-xylose reductase (XR, NCU08384), xylitol dehydrogenase (XDH, NCU00891), L-arabitol dehydrogenase (LAD NCU00643) and L-xylulose reductase (LXR NCU09041). The growth phenotypes showed that the deletion of *xr* or *xdh* significantly affected the growth on either D-xylose or L-arabinose. The mutants of *xr*, *lad*, or *lxr* led to a reduced mycelia density and fewer conidia on L-arabinose (Figure 2). The results indicate that D-xylose reductase accounts for the majority of the reductase activity on both L-arabinose and D-xylose in *N. crassa*, which is similar to the situation in *T. reesei*[25].

### Transcriptome analysis of *N. crassa* growth on D-xylose, L-arabinose versus D-glucose

The transcriptome of *Neurospora crassa* growing on three monosaccharides (D-glucose, D-xylose and L-arabinose) was conducted using the RNA-sequencing technique. Clean reads were mapped to the *N. crassa* genome sequence and reads per kilobase of exon model per million mapped reads (RPKMs) were calculated as the normalized expression values of each annotated gene. Differential expression analysis was conducted using the DESeq [27] (Additional file 1: Table S1). High reproducibility of the RNA-seq data from two biological replicates was observed under both D-glucose and L-arabinose conditions, with a Spearman correlation coefficient above 0.9 and *P*-value less than 0.001 and the averaged RPKMs were use for subsequent analysis (Additional file 2: Figure S1). To discover significantly upregulated and downregulated genes between the conditions tested, only the genes with *P*-values of DESeq analysis less than 0.001 and RPKM values above 20 in at least one condition went into further analysis. These criteria identified genes with robust differences in expression [28]. Using gene expression on D-glucose as a reference, 1,046 genes on L-arabinose and 105 genes on D-xylose showed significantly differential expression. In total, 498 genes were upregulated on L-arabinose, in contrast to only 96 genes upregulated on D-xylose, with 50 genes upregulated on both sugars (Table 1). These data clearly suggested there was a much wider response in *N. crassa* to L-arabinose than that to D-xylose. The difference was surprising, since D-xylose and L-arabinose are catabolized via the same metabolic pathway after xylitol is formed and only two more genes are needed based on the catalytic pathway predicted for L-arabinose (LAD and LXR, as discussed previously).

**Table 1:** The number of genes showing differential expression on L-arabinose and D-xylose vs D-glucose

	**L-arabinose**	**D-xylose**	**L-arabinose and D-xylose**
Upregulated	498	96	50
Downregulated	548	9	8

### Identification of genes induced by both D-xylose and L-arabinose

**Figure 3:**
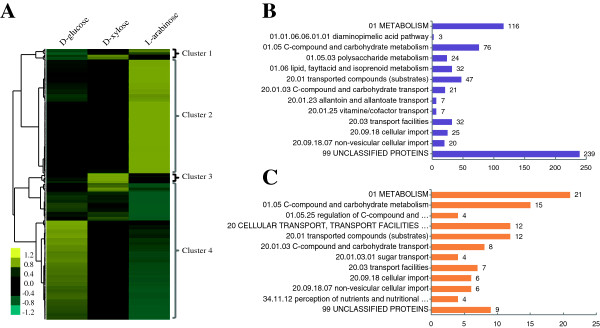
**Hierarchical clustering analysis and functional category analysis of RNA-seq data. (A)** Hierarchical clustering analysis of genes with values more than 20 for reads per kilobase of exon model per million mapped reads, showing significantly differential expression in *Neurospora crassa* when grown on L-arabinose or D-xylose compared to that on D-glucose. **(B)** and **(C)** functional category analysis of genes grouped into cluster 2 and cluster 3.

In order to elucidate the transcriptional data more clearly, we performed hierarchical clustering analysis of genes that showed significant differential expression on L-arabinose or D-xylose, compared to D-glucose as the reference. The analysis used Cluster 3.0 [29] with euclidean distance as the similarity metric and complete linkage as the clustering method, and resulted in four distinct clusters (Figure 3A and Additional file 3: Table S2). The 45 genes induced by both L-arabinose and D-xylose similarly, compared to D-glucose, were grouped into cluster 1, in which the genes involved in C-compound/carbohydrate metabolism and transport were enriched based on function category analysis (Additional file 3: Table S3). This cluster included three hemicellulase genes (NCU01900, *gh43-2*; NCU07225, *gh11-2*; NCU09652, *gh43-5*), three sugar transporters (NCU00801, NCU07607, NCU05897), two genes involved in the pentose catabolic pathway (NCU08384, *xr*; NCU00891, *xdh*) and xylan degradation regulator 1(*xlr-1*, NCU06971).

### Genes induced by L-arabinose but not by D-xylose

The second cluster (cluster 2) contained 460 genes that showed relative higher expression levels on L-arabinose, but not on D-xylose. Function category analysis [30] showed that the majority of these genes represent unclassified proteins (Figure 3B and Additional file 3: Table S4). Apart from these unclassified genes, C-compound/carbohydrate metabolism and transport genes were the most enriched group (*P*-value <1E-9), as expected. A total of 21 carbohydrate transport genes were induced, including multiple identified sugar transporters: NCU00821 (*Xyp29*, D-xylose-specific transporter), NCU08114 (*cdt-2*, cellodextrin transport) and NCU02582 (*rco-3*, glucose transporter) [9,31,32], and 10 putative sugar transporters, which will be discussed later. In addition, 50 of 300 CAZy genes were significantly and highly upregulated when exposed to L-arabinose compared to their expression on D-glucose (Additional file 4: Table S6), of which 46 were grouped into this cluster. These genes included five hemicellulase genes, of which two hemicellulase genes with RPKM values more than 700, NCU08189 (endo-1,4-beta-xylanase) and NCU02343 (alpha-L-arabinofuranosidase), showed expression levels that were increased over 250-fold. In addition, this cluster contained three of five beta-galactosidases in the *N. crassa* genome (NCU04623, NCU00642, NCU00810), with average RPKM values more than 130.

A smaller cluster of 40 genes (cluster 3) showed high expression levels in *N. crassa* when exposed to D-xylose, but were not induced by L-arabinose. This group contained two exoglucanases (NCU09680 and NCU07340) with an average RPKM value of 31 and one gene encoding an enzyme of the pentose catabolism pathway (NCU11353, D-xylulose kinase), consistent with a previous study using microarray analysis [8]. Not surprisingly, the carbohydrate metabolism and transport group were enriched, although the number of genes was less than that induced by L-arabinose (Figure 3C and Additional file 3: Table S5). Four putative sugar transporters were induced significantly by D-xylose, about a third of the number induced by L-arabinose; detailed analysis of sugar transporter gene expression will be discussed separately later.

### The L-arabinose regulon in *Neurospora crassa* was significantly different and wider than that of the carbon starvation response

In addition to unregulated genes, there are also a large number of genes showing downregulated expression levels on L-arabinose compared to the D-glucose (cluster 4). The majority of genes were involved in the metabolism of amino acids, carbohydrates, nitrogen and nucleotides, including a key enzyme for the tricarboxylic acid (TCA) cycle (NCU01227, oxoglutarate dehydrogenase) and two critical genes in the glycolysis pathway (NCU06075, Pyruvate kinase; NCU00629, 6-phosphofructokinase); no such decrease under D-xylose conditions was observed. These differences indicate that L-arabinose is a less desirable carbon resource compared to both D-glucose and D-xylose, and that it may result in the induction of the carbon starvation response [33] We performed a comparison of the L-arabinose regulon (498 upregulated and 548 downregulated genes) with carbon starvation responses, using RNA-seq data from cells grown with no carbon source [34]. The Venn diagrams showed that the overlap of the response under both conditions was 177 genes showing increased expression and 276 genes showing decreased expression levels respectively (Additional file 5: Figure S2 and Additional file 6: Table S7). Despite this high overlap, there were also clear differences between the two conditions. A total of 321 genes, nearly twice the number showing overlap, showed significantly induced expression only by L-arabinose. These genes were mainly grouped into polysaccharide metabolism, the pentose-phosphate pathway and C-compound and carbohydrate transport. The genes responding only to carbon starvation were mainly involved in amino acid metabolism, complex cofactor/cosubstrate/vitamin binding and energy metabolism. In addition, eight hemicellulase genes showed increased expression with an average RPKM value of 344, and five hemicellulase genes responding to carbon starvation had an average expression value of 52. These data suggested that genes induced by L-arabinose were not solely due to carbon starvation, although the carbon starvation process was launched as part of the L-arabinose response.

Among the gene set downregulated by L-arabinose, however, the overlap was larger, with 276 genes showing reduced expression on both L-arabinose and no carbon, and 272 genes downregulated only in L-arabinose. The functions of the 276 genes downregulated in both conditions were related to energy, nucleotide/nucleoside metabolism and protein synthesis. Since the fungus grows much slower on these conditions compared to D-glucose and D-xylose, the metabolic pathways may be slowed down to save energy, a strategy that was also found during growth on Avicel [33].

Together, the observation that L-arabinose induced a much broader spectrum of genes than D-xylose, indicated the L-arabinose might be acting not only as a carbon source, but also as a signal molecule for less desirable carbon. Such a signal could regulate core metabolic switches and further rewire the whole metabolic network, including provoking many pathways of gene expression, such as sugar transporters and hemicellulases.

### Sugar transporter expression of *N. crassa* grown on D-xylose, L-arabinose and D-glucose

Multiple strategies have been taken to improve D-xylose and L-arabinose fermentation through *S. cerevisiae* engineering. Using the pentose transporter to enhance pentose uptake might be a promising complementary solution with metabolic engineering [11,35-37]. Only a few pentose transporters have been identified so far, with limited improvement for pentose fermentation [37-39].

Therefore, the discovery of novel pentose transporters is required. Based on the annotation of TransportDB database [40] and the *N. crassa* database (Broad Institute), a total 39 genes are predicted as putative sugar transporters belonging to the major facilitator superfamily (MFS) in the *N. crassa* genome (Table 2). Among these 39 putative sugar transporter genes, 24 genes had transcripts that were expressed (RPKM ≥20) on L-arabinose, whereas 15 were expressed on D-xylose and 6 genes on D-glucose.

**Table 2:** **The expression of predicted sugar transporter genes in the genome of *Neurospora crassa *** **on D-glucose, L-arabinose or D-xylose**

**Locus**	**Annotation**	**Reads per kilobase of exon model per million mapped reads (RPKM)**		
		**D-glucose**	**L-arabinose**	**D-xylose**
NCU01633	Hexose transporter HXT13	**242.45**	9.92	29.68
NCU02188	Sugar transporter	1.20	**1200.18**	4.43
NCU08114	Hexose transporter	1.24	**587.22**	**138.64**
NCU09287	Sugar transporter	1.55	**560.93**	30.21
NCU00821	Sugar transporter	32.87	**414.86**	57.54
NCU10021	MFS monosaccharide transporter	6.61	**394.47**	**1045.02**
NCU00809	MFS monosaccharide transporter	7.07	**314.73**	6.64
NCU01132	MFS monosaccharide transporter	0.46	**280.94**	0.87
NCU06138	Quinate permease	0.74	**213.95**	75.43
NCU05897	L-fucose permease	1.10	**179.47**	**315.45**
NCU05853	MFS sugar transporter	2.36	**149.95**	5.65
NCU04963	High-affinity glucose transporter	4.91	**128.69**	**402.90**
NCU04537	Monosaccharide transporter	1.06	3.35	**410.00**
NCU05627	High affinity glucose transporter ght1	4.47	48.96	**244.36**
NCU00450	Sucrose transporter	28.78	52.91	23.15
NCU00801	MFS lactose permease	6.10	25.15	24.96
NCU00988	MFS quinate transporter	0.18	47.98	0.86
NCU01494	MFS sugar transporter	1.34	6.47	2.07
NCU01813	High affinity glucose transporter	23.68	38.96	16.02
NCU01868	MFS maltose permease malp	0.41	2.42	0.54
NCU02582	Rco3	9.65	42.90	12.30
NCU04310	Sugar transporter	9.68	26.59	12.88
NCU05350	Sugar transporter	0.37	10.25	0.55
NCU05585	MFS quinate transporter	1.01	47.89	1.52
NCU06026	Quinate permease	5.99	37.83	13.25
NCU06358	High affinity glucose transporter RGT2	1.71	51.82	18.50
NCU06384	MFS sugar transporter	0.08	2.88	1.11
NCU06522	MFS maltose permease	1.35	3.39	1.98
NCU07054	Sugar transporter 4	0.03	0.41	0.14
NCU07169	MFS glucose transporter	13.55	30.96	13.49
NCU07607	Sugar transporter	2.53	10.65	23.97
NCU07861	Maltose transporter	0.54	1.05	0.67
NCU08152	High affinity glucose transporter	0.05	0.71	0.09
NCU08180	High-affinity glucose transporter	0.00	0.01	0.00
NCU08858	MFS alpha-glucoside transporter	24.76	36.86	37.86
NCU09321	Sucrose transporter	33.00	37.66	35.30
NCU09358	Hexose carrier protein	0.27	15.90	0.77
NCU11342	MFS hexose transporter	0.15	1.51	0.63
NCU12154	Maltose permease MAL61	2.10	2.59	1.51

The genes with RPKM values more than 100 are shown as bold font.

A total of 14 transporters showed high expression levels with RPKM values of more than 100, and none of these showed high induction on glucose and both pentose sugars, indicating their expression was very specifically regulated. Only NCU01633 showed D-glucose-specific induction whereas 13 genes were specifically induced by D-xylose or L-arabinose. Based on previous publications, NCU00821 (*An25*) encodes a D-Xylose-specific transporter [9], whereas NCU00809 and NCU08114 (CDT-2) have the capability of transporting cellodextrin [32]. NCU10021 (Hgt*-*1) was identified as a glucose transporter [41]. The remaining ten transporters had not been well-characterized so far, although limited analysis has been performed on five of them; NCU04963 and NCU06138 have the capability to transport both xylose and glucose, whereas very weak xylose transportation activity was detected in a recombinant yeast strain harboring NCU02188, NCU04537 or NCU09287 [9]. We decided to test the substrates of five novel predicted transporters NCU01132, NCU01633, NCU05627, NCU05853, NCU05897, using heterologous expression of each transporter in the hexose-null yeast strain EBY.VW4000.

### GLT-1, a novel D-glucose transporter identification

Of the five engineered yeast strains expressing putative sugar transporters, only NCU01633 enabled the cells to grow efficiently on D-glucose; the recombinant strain expressing NCU05627 has very limited growth (Additional file 7: Figure S3A) and no growth could be detected of recombinant strain with NCU01132, NCU05853 and NCU05897 on D-glucose (data not shown). These results are consistent with the transcript expression results on D-glucose for these genes. We further conducted uptake assays of recombinant yeast expressing NCU01633 for D-glucose, D-xylose and L-arabinose using radioactively labeled sugars in final concentrations of 3 mM. The results showed that D-glucose uptake was two orders of magnitude higher compared to that of D-xylose with no detectable transport activity for L-arabinose (Additional file 7: Figure S3B). We conclude that NCU01633 is a D-glucose transporter of *N. crassa*, which was therefore named as D-glucose transporter 1 (GLT-1).

### Two novel pentose transporters identification and characterization

The remaining four transporter genes showed really different performances in the monosaccharide uptake assays. The recombinant yeast strains with NCU05853 or NCU05897 did not transport either pentose sugar, although they were highly induced by D-xylose or L-arabinose. Since both genes were also induced significantly on the Avicel condition and no carbon source condition, it suggested those two transporters were not pentose transporters and that their induction on L-arabinose might be part of the response to carbon starvation.

**Figure 4:**
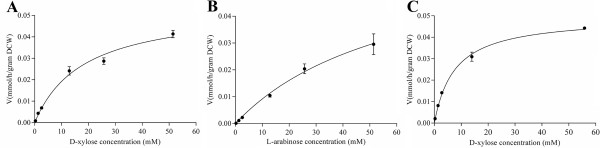
**Kinetics of xylose transporter-1 (XAT-1) for D-xylose (A) and L-arabinose (B) and the kinetics of XYL-1 for D-xylose (C).** The rate of sugar transport was determined as a function of sugar concentration. The transport rate was normalized for yeast dry cell weight (DCW). The background values determined with cells containing the empty vector were subtracted. Error bar indicates the standard deviation of the triplicates.

However, NCU01132 can transport both D-xylose and L-arabinose whereas NCU05627 transports D-xylose, and the kinetics of pentose transport by both overexpression strains were examined further. The Km value of NCU01132 for L-arabinose was 61.93 ± 17.68 mM and the corresponding Vm was 65.84 ± 11.76 μmol/h/gram dry cell weight (DCW) (Figure 4A). However, Km and Vm for D-xylose were 18.17 ± 3.23 mM and 54.11 ± 3.83 μmol/h/gram DCW respectively (Figure 4B), which indicated the uptake activity and affinity of NCU01132 for D-xylose was higher than that for L-arabinose, despite NCU01132 showing higher transcript levels on L-arabinose. Based on these data, we conclude that NCU01132 encodes a transporter of D-xylose and L-arabinose, which was named XAT-1. For NCU05627 expressed in the EBY VW4000 strain, the cells mediated D-xylose uptake with Km and Vm values of 7.58 ± 0.60 mM and 49.61 ± 1.20 μmol/h/gram DCW respectively (Figure 4C), whereas it had an undetectable uptake rate for L-arabinose. It was therefore named xylose transporter 1 (XYT-1). Both XAT-1 (NCU01132) and XYT-1 (NCU05627) showed higher substrate affinity compared with the previously discovered D-xylose-specific transporters An25 and Xyp29 [9] and suggest these might be good candidates for engineering xylose fermentation in *S. cerevisiae* in the future.

### HCR-1 encodes a novel transcription factor involved in regulation of hemicellulase expression on L-arabinose and xylan

**Figure 5:**
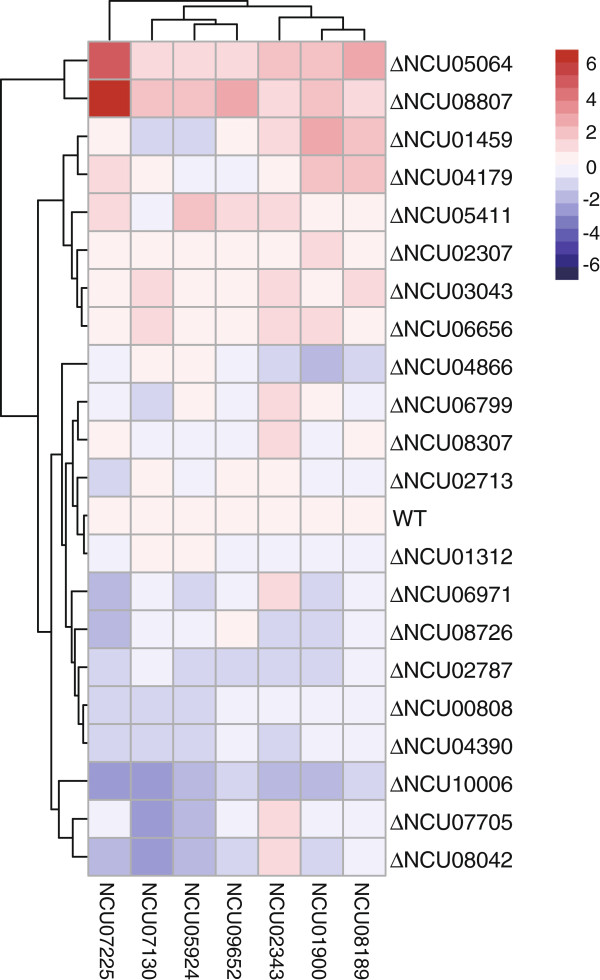
**Hierarchical cluster analysis of relative transcript levels of hemicellulase genes in the transcription factor deletion strains versus the wild-type strain monitored by quantitive real time-PCR after exposure to L-arabinose for 4 h.** Data are shown as a heat map with the following color code of relative expression values (navy: 6; white: 0; firebrick: -6; numbers indicate the log2 of the relative expression level, n = 2). Strains were precultured in 2% D-glucose for 16 h, washed, and transferred into minimal medium with 2% L-arabinose for an additional 4 h. Transcript levels of hemicellulase coding genes were assessed relative to those in the wild-type strain within each experiment. The experiment used actin as the internal standard. The values given in the figures are the means of three independent experiments.

**Figure 6:**
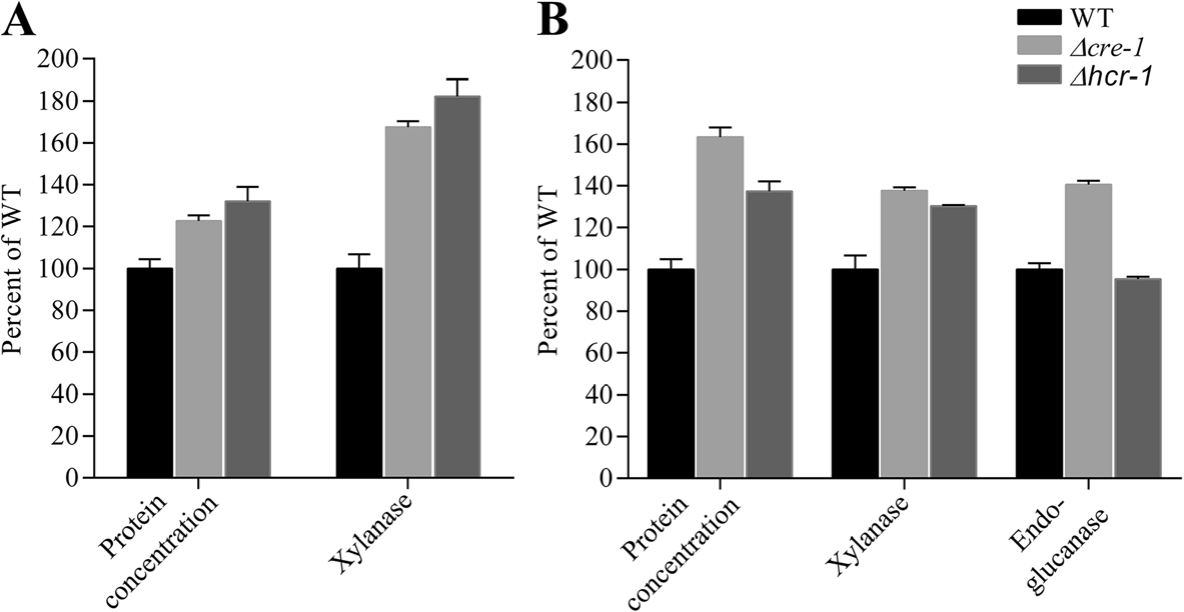
**Protein concentration, xylanase activity and endoglucanase activity of culture filters from *N. crassa *wild-type (WT), **Δ***hcr-1 *and** Δ***cre-1 *strains cultivated at 25°C for 7 days on 2% beechwood xylan (A) and 2% Avicel (B).** The biomass production was similar in the WT and the Δ*hcr-1* mutant on both conditions.

In *T. reesei*, L-arabinose and D-xylose are able to induce xylanase expression, which is fully dependent on the regulator XYR1 [16]. The L-arabinofuranodiase was reduced by disruption of regulator AraR in *A. niger* on L-arabinose [18]. In our transcriptome data of *N. crassa*, there were eight hemicellulase genes significantly induced by L-arabinose (the average RPKM was 429), whereas D-xylose induced five hemicellulase genes with an average RPKM value of 199, much lower than that on L-arabinose. Impressively, there were 21 transcription factors significantly upregulated (*P*-value <0.001 and RPKM values >20) during L-arabinose growth, including *xlr-1* and *clr-1* (Additional file 8: Table S8), whereas only two (NCU06971, *xyr-1*; NCU02142) showed differential expression levels on D-xylose (using the expression on the D-glucose condition as reference). In order to test whether any of those transcription factors were involved in the regulation of hemicellulase expression upon L-arabinose, we checked the relative expression levels of seven classical hemicellulase genes in deletion mutants of these transcription factors versus WT strains exposed to L-arabinose. Of 21 L-arabinose-induced transcription factors, 19 were available in the Fungal Genetics Stock Center (FGSC) as knock-outs, and were tested using the *cre-1* and *clr-2* mutants as reference. The strains were pre-grown on D-glucose for 16 h, washed and then transferred into 2% L-arabinose for an additional 4 h before the transcript levels of hemicellulase genes were assessed by quantitative real-time PCR. Expression of the seven hemicellulase genes showed up- or downregulation in multiple transcription factor mutants (Figure 5 and Additional file 8: Table S9). However, the expression pattern in mutants of NCU05064 was particular interesting. Transcripts of all seven tested hemicellulase genes showed significant increases in the NCU05064 deletion strain, with increases comparable to the *cre-1* mutant. The data suggest the NCU05064 might be a novel hemicellulase expression suppressor. In order to test our hypothesis, we further checked the hemicellulase expression in mutants of NCU05064 on xylan and Avicel with the *cre-1* mutant strain as the reference. Although the biomass growth of the mutant was similar to that of the WT strain (Additional file 9: Figure S4), the secreted protein and hemicellulase/xylanase activity showed obvious increases in the NCU05064 knock out strain in both conditions, with changes comparable to the *cre-1* deletion strain (Figure 6). However, the cellulase activity (endoglucanase) was not affected significantly in this mutant. Together, the results suggest that we have identified a novel hemicellulase regulator, which was named HCR-1. The detailed regulatory network of HCR-1 will be addressed in future.

### L-arabitol induces hemicellulase genes more efficiently than L-arabinose in *N. crassa*

**Figure 7:**
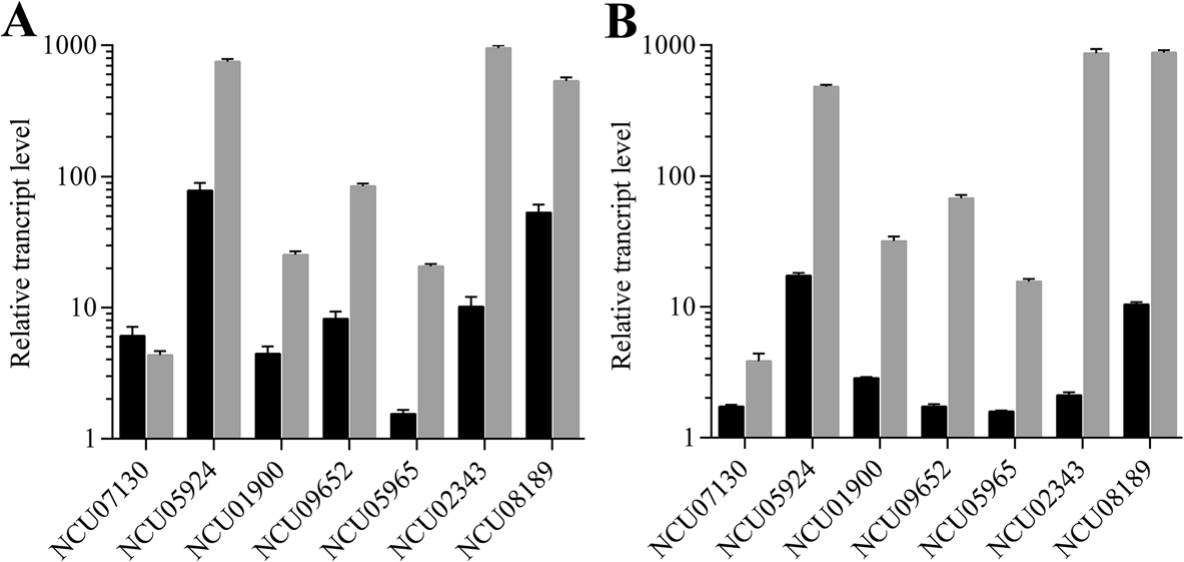
**The transcript levels of hemicelluloses induced by L-arabinose and L-arabitol in *N. crassa *wild-type stain (A) and *xr *deletion (B) strain.** The wild-type strain and *xr* mutant strain were precultured in Vogels’ minimal medium with 2% D-glucose for 16 h, washed and transferred to minimal medium supplemented with 2% L-arabinose or 2% L-arabitol for an additional 4 h. The expression levels of hemicellulase genes were monitored by quantiative real time-PCR, after growth on L-arabinose (light grey bars), or L-arabitol (light black bars). Transcript levels were assessed relative to gene expression in minimal medium with 2% D-glucose within each experiment. The experiment used actin as the internal standard. The values shown are the means of three independent experiments.

Hemicellulase genes show higher expression on L-arabitol than that on L-arabinose in *T. reesei*[42]. In order to assess whether xylanase induction by L-arabitol is more efficient than by L-arabinose in *N. crassa*, quantitative RT-qPCR was performed (Figure 7). Six of seven tested hemicellulase genes showing higher expression levels induced by L-arabitol than L-arabinose, except NCU07130 (Endo-beta-1,4-xylanase, GH10). This result suggests that L-arabitol is the better inducer in both *T. reesei* and *N. crassa* and that this preference might be conserved in other filamentous fungi. Since L-arabitol was formed from L-arabinose via D-xylose reductase in filamentous fungi, we performed the same assay in the *xr* (NCU08384, D-xylose reductase) deletion strain. The results showed that the hemicellulase genes were induced much less than WT, but still could be induced significantly. These data suggest that for hemicellulases in *N. crassa*, there is an alternative way for hemicellulase induction from L-arabinose rather than through D-xylose reductase in *N. crassa*.

## Discussion

The utilization of all three sugars, D-glucose, D-xylose and L-arabinose is essential for the economical conversion of plant biomass into fuels and chemicals. A variety of filamentous fungi can grow on pentose, including *N. crassa*. The pentose-utilization fungal genomes are a good resource for mining novel gene elements, such as D-xylose transporters for D-xylose fermentation metabolic engineering in *S. cerevisiae*. As important industrial microbes, the improvement of filamentous fungi through metabolic engineering attracts more and more interest, including the production of enzymes and chemicals using pentose sugars as the sole carbon source and inducers [43,44]. Both demands require a deep understanding of the pentose utilization pathway in filamentous fungi, not only for D-xylose but also for L-arabinose. Compared with what has been known about D-xylose utilization and the genome-wide analysis of microbe responses to it [4,6-8,14], very little is known about that of the L-arabinose. The transcriptome analysis of filamentous fungi on L-arabinose has not been reported, to our knowledge. Taking advantage of *N. crassa* as a model organism and the RNA-seq technique, the transcriptome of the fungus cell grown on D-xylose, L-arabinose or D-glucose were analyzed. Although only two extra genes are theoretically needed for L-arabinose metabolism compared to the D-xylose, the genome-wide response to L-arabinose was dramatically different to that for D-xylose in *N. crassa*. In addition, 50 CAZy genes showed highly and significantly increased expression levels on L-arabinose compared to those on D-glucose, including 8 of the 19 predicted hemicellulase genes with an average RPKM value of 363. It has been reported that L-arabinose is very good inducer of xylanase production in *T. reesei* Rut C-30 and that the highest total xylanase activities were achieved when cells use L-arabinose as the main carbon source compared to the xylose [45,46]. However, the expression of some pentose phosphate pathway and TCA cycle genes were downregulated, while the genes involved in sugar transport, polysaccharide metabolism and lipid, fatty acid metabolism were significantly upregulated on L-arabinose. From the present study, L-arabinose has a really different impact on *Neurospora crassa* compared with D-xylose. It may not only serve as a less-desired carbon source, but might also be a signal molecule, which could rewire the whole metabolic pathway and provoke much wider and deeper transcriptional response than that of D-xylose in this fungus. Although the fungus response to L-arabinose had significant overlap with carbon starvation (no carbon condition) [34], especially the downregulated genes, there were significant differences and a wider range of gene induction was found by L-arabinose compared to carbon starvation, particularly the sugar transporters, hemicellulase genes and transcription factors. Whether this response is conserved in other filamentous fungi, such as *Trichoderma* and *Aspergilli*, needs transcriptome data on L-arabinose for those fungi.

Sugar transporters are a critical component of the sugar metabolic pathway. Based on our transcriptome data, surprising numbers of sugar transported were induced by L-arabinose, much more than D-xylose, D-glucose and even Avicel and xylan. This difference cannot be explained as carbon starvation, as multiple sugar transporters were expressed much more highly in L-arabinose than that in the no carbon condition. A wide variety of sugar transporters, including those for the polysaccharide celludextrin, xylose, and even glucose transporters all were provoked, which clearly suggests that L-arabinose might be a pivotal signal for sugar sensing and metabolism in *N. crassa* in the natural environment for plant biomass degradation. In addition, the transcriptome data of the fungus upon three monosaccharides generated in the present study provide good resources to identify novel sugar transporters. The GLT-1, XAT-1, XYT-1 for D-glucose transporter, D-xylose transport and L-arabinose transport are examples that were characterized further here. During this manuscript preparation, the NCU02188 was identified as L-arabinose transporter [33], consistent with our expression data, which showed that LAT-1 was the highest transcribed gene in *N. crassa* on L-arabinose. Our preliminary results have shown that the recombinant yeast strain with LAT-1 does complement growth of EBY.VW4000 strain on D-glucose, indicating this protein can transport D-glucose also. However, the L-arabinose uptake rate was much higher than that of D-glucose (Additional file 7: Figure S3). Thus, LAT-1, with the other two pentose transporters identified in present study, XAT-1 and XYT-1, were good candidates for pentose fermentation yeast engineering in the future.

L-arabitol is known to induce the expression of xylanases more efficiently than L-arabinose in *T. reesei*[42]. This preference is conserved based on our data, with L-arabitol also showing better xylanase induction than L-arabinose in *N. crassa*. However, we still see some induction by L-arabinose in the deletion strain of D-xylose reductase (NCU08384), which should be unable to convert L-arabinose to L-arabitol, suggesting that either L-arabinose itself or its derivatives other than L-arabitol can also induce the hemicellulases in *N. crassa*. A similar observation has been reported in *T. reesei*[47]. There were 21 transcription factors with high expression values that were upregulated in L-arabinose conditions. Taking advantage of the stock of the gene knock outs, a novel C2H2 family transcription factor (NCU05064 named as HCR-1) was shown to be involved in hemicellulase regulation by both L-arabinose and xylan. Interestingly, after the deletion of *hcr-1*, the secreted protein and xylanase activity were increased, compared to the WT strain, but the endoglucanase activity was similar to the WT strain after 7 days on the Avicel condition (Additional file 6: Figure S2). These results suggest that HCR-1 is a hemicellulase-specific regulator. Through phylogenetic analysis, the HCR-1 homologues were found in a variety of cellulolytic fungi (Additional file 10: Figure S5), suggesting that the roles of the HCR-1 might be conserved in fungi. The detailed mechanism and the regulatory network of HCR-1 need more experiments in the future, for example, to determine whether HCR-1 has any interactions with the known ligonocellulase regulators, such as XLR-1(NCU06971), CRE-1, CLR-1, CLR-2, and so on.

The transcriptome comparison analysis in the present study provides a first glimpse of the genome-wide profiles of *N*. on L-arabinose, and is also the first transcriptional profile comparison of fungi grown on the three most abundant lignocelluloses monosaccharides. The identification of a large L-arabinose regulon in present study sheds much light on the molecular basis of filamentous fungi response to this pentose. Finally, a group of novel sugar transporters, especially two novel pentose transporters identified from the present study, would be helpful in the future to manipulate for hexose-pentose co-fermentation engineering in industrial microbes, such as *Saccharomyces cerevisiae.*

## Conclusions

This study provides the first transcriptomic comparison analysis of *N. crassa* exposed to L-arabinose versus D-xylose and D-glucose. The dramatic differences of gene expression profiles were found in *N. crassa* exposed to D-xylose and L-arabinose. The much deeper and wider response to L-arabinose was detected in this fungus. The present study deepens the understanding of the utilization of L-arabinose and D-xylose in filamentous fungi, and has discovered novel sugar transporters (GLT-1, XAT-1 and XYT-1) and a hemicellulases regulator (HCR-1) that are good targets for metabolic engineering in both yeast and filamentous fungi for enzymes and cellulosic biofuels production.

## Methods

### Strains and growth conditions

The *N. crassa* WT strain (FGSC2489) and all gene deletion strains were obtained from FGSC and the mutants were confirmed by PCR with a specific primer for the target gene and a common primer for the hygromycin cassette. The strains were cultivated in slant culture medium with Vogel’s minimal medium [48] supplemented with 2% sucrose (Amresco, Solon, USA) at 28°C under constant light.

For transcriptome analysis (RNA-Seq), 10^8^ conidia from 10-day-old slants were collected and inoculated into 100 ml media (1 × Vogel’s salts with 2% carbon source (D-glucose, D-xylose or L-arabinose) in 250-ml flask, then grown for 16 to 26 h at 25°C, with shaking at 200 rpm under constant light. Mycelia were harvested by vacuum filtration with Whatman filter paper, frozen immediately in liquid nitrogen and stored at -80°C for RNA extraction.

In the transfer experiment, all the strains were pre-grown for 16 h on 2% glucose and transferred to Vogel’s salt supplemented with appropriate sugar as the sole carbon source, except for sugar preference analysis in Figure 1A-C, in which mixed sugars of two monosaccharides were used as carbon sources after mycelia transferring. For RT-qPCR analysis, the transferred mycelium was incubated for additional 4 h at 25°C under constant light, before harvested, frozen in liquid nitrogen and stored at -80°C. D-glucose, D-xylose and L-arabinose were purchased from Sigma-Aldrich (St Louis, MO, USA).

*S. cerevisiae* EBY.VW4000 [49] was cultured in YPM medium (1% yeast extract, 2% peptone and 2% maltose) at 30°C and shaken at 250 rpm for aerobic growth. *S. cerevisiae* strains harboring plasmids were grown in synthetic drop-out medium (0.67% yeast nitrogen base, 0.2% amino acid drop out mix and 2% maltose), supplemented with amino acids except the nutrient of the plasmid marker. *Escherichia coli* DH5α was used for plasmid amplification and vector construction and cultivated in Luria-Bertani medium with antibiotics according to plasmid selection.

### RNA extraction

Total RNA from frozen samples was isolated using Zirconia/Silica beads (0.5 mm diameter; Biospec) and a MiniBeadbeater (Biospec, Bartlesville, USA) with 1 mL TRIzol reagent (Invitrogen Life Technologies, Carlsbad, CA, USA) according to the manufacturer’s protocol. An additional clean-up was performed to eliminate genomic DNA contamination using the RNeasy mini kit (Qiagen, Hilden, Germany), according to the manufacturer’s RNA clear-up instructions. Nanodrop and agarose gel electrophoresis were used to check RNA integrity and concentration [50].

### RNA sequencing and data analysis

The purified RNA samples, with RIN (RNA Integrity Number) over 8.0, determined by Agilent 2100 Bioanalyzer (Agilent Technologies, Waldbroon, Germany), from mycelia cultivated with D-glucose, L-arabinose or D-xylose as the sole carbon source were sequenced at Beijing Genomics Institute (Shenzhen, China), who constructed their digital gene expression libraries and sequenced by means of Illumina HiSeq™ 2000 platform to obtain the expression libraries of 49-nt read length. Prior to analyzing the data, the quality of raw sequencing reads was checked by the FastQC tool (v0.10.0) [51] and low quality and dirty reads with sequence adaptors, more than 20% QA <25 bases or 'N’ bases were removed using NGSQCToolkit (v2.3) [52].

The clean reads were mapped against predicted transcripts from the *N. crassa* OR74A genome [53] (version 12) with less than two-base mismatching, using Tophat (version 2.0.8b) [54]. The alignment results were stored in SAM format files for subsequent analysis. The counts of reads that uniquely mapped to only one gene were calculated for each gene by HTseq-count (http://www-huber.embl.de/users/anders/HTSeq) using SAM files and genome annotation as input. The normalized expression values for each gene were calculated by the number of uniquely mapped RPKM and those genes having RPKM values more than 20 were defined as the highly expressed genes [28]. Independent duplicate cultures were sampled and inter-sample correlation comparison was analyzed for *N. crassa* on D-glucose and L-arabinose (Spearman correlation coefficient >0.9 and *P*-value <0.001, see Additional file 2: Figure S1). Differential expression gene was determined by DESeq package (v1.5.1) [27] with raw counts of reads mapping to unique genes as input. The transcript levels of genes having a *P*-value of less than 0.001 in the L-arabinose or D-xylose sample compared to that in the D-glucose sample, were considered as showing significant differential expression. Profiling data are listed in Additional file 1: Table S1 and the sequence data produced in this study can be accessed [GEO: GSE51091]. The RNA-seq data of *N. crassa* cultivated on medium for 4 h with no carbon source was downloaded from the GEO database [GEO: GSE35227] generated in previous study [34].

### Quantitative real time-PCR

Quantitative PCR was performed using a CFX96 real-time PCR detection system (Bio-Rad, Hercules, USA) with reagents from TOYOBO (One-step qPCR Kit, OSAKA, Japan). The PCR reaction mixture (20 μl) with three replicates included 75 ng template RNA, 0.4 μM primers and 10 μl RNA-direct SYBR® Green Realtime PCR Master Mix, according to the manufacturer’s instruction. The relative transcript level of each gene was calculated by method of 2^-ΔΔCt^ with its expression in the WT as the control and the expression of actin (NCU04173) as an internal standard.

### Plasmid and strain construction

RNA samples from *N. crassa* mycelia were reverse transcribed and used as the template for amplification of genes encoding putative pentose transporters. The plasmid pRS426 with phosphoglycerate kinase-1 promotor, green fluorescent protein open reading frame and CYC1 terminator was used as the shuttle vector. The putative sugar transporter genes were amplified, cloned into the shuttle vector and then transformed into *E. coli* DH5α and plated on LB medium containing 100 mg/L ampicillin. Single colonies of transformants were cultured in LB liquid medium supplemented with 100 mg/L ampicillin and plasmids was checked by PCR and restriction digestion, after extraction from transformants. All target genes cloned into shuttle vectors were sequenced to verify the authenticity of the plasmid construction and yeast transformations were performed as described previously [55].

### Sugar uptake analyses

Sugar transport assays were performed using a modification of the method described previously [9,56]. In order to determinate the uptake kinetics of L-arabinose and D-xylose, an aliquot (100 μl) of yeast strains with an optical density (600 nm) of 50 was suspended in 100 mM Tris-citrate buffer at pH 5.0 was incubated at 30°C for 5 minutes, mixed with 50 μl of a sugar solution containing (1-^3^H)-labeled L-arabinose or (U-^14^C)-labeled D-xylose (American Radiolabeled Chemicals Inc., St Louis, MO, USA) for 2 minutes at 30°C and 1 ml ice-cold water was added to stop the reaction. After centrifugation (10,000 rpm, 1 minute), the cells were washed three times with ice-cold water, mixed with 1 ml 1 M NaOH and then transferred to 5 ml of scintillation cocktail (Thermo scientific). The radioactivity was measured by scintillation counter (Tri-Carb 2910TR Liquid Scintillation Analyzer, PerkinElmer, US). An aliquot of 300 μl of each yeast strain was dried at 80°C for determining the DCW. The results shown were the average results of at least two independent experiments.

### Enzyme activity measurements

The strains were cultured at 1 × Vogel’s salts with 2% carbon sources at 25°C for 7 days under constant light. Total secreted protein in supernatants was determined by using a Bio-Rad DC protein assay kit (Bio-Rad, Hercules, USA). Endoxylanase activity in culture supernatants of *N. crassa* strains grown on 2% beechwood xylan (Beechwood, Sigma, St Louis, MO, USA) or Avicel (PH 101, Fluka, St Louis, MO, USA) medium for 4 days and 7 days was measured, according to the method described previously [8]. Endoglucanase activity on azo-carboxymethyl cellulose (azo-CMC) in culture supernatants of *N. crassa* exposed to Avicel was determined via the azo-cm-cellulose assay kit (Megazyme; S-ACMC, Bray, Co. Wicklow, Ireland), in accordance with the manufacturer’s directions.

### Phylogenetic analysis

Putative orthologs to HCR-1 in selected fungal species were identified as best reciprocal BLAST hits from the National Center for Biotechnology Institute (NCBI) protein database. The phylogenetic trees were inferred using the Neighbor-Joining method. The tree is drawn to scale, with branch lengths in the same units as those of the evolutionary distances. The evolutionary distances were computed using the Poisson correction method and evolutionary analyses were conducted in MEGA5. The strains and NCBI accession numbers used in each tree were as follows: *Aspergillus terreus* NIH2624 (XP_001213684.1), *Aspergillus fumigatus* A1163 (EDP52766.1), *Aspergillus oryzae* RIB40 (XP_001818207.1), *Trichoderma reesei* QM6a (EGR48170.1), *Aspergillus niger* ATCC 1015 (EHA21959.1), *Trichoderma virens* Gv29-8 (EHK19061.1), *Trichoderma atroviride* IMI 206040 (EHK39892.1) *Myceliophthora thermophila* ATCC 42464 (XP_003665650.1), *Thielavia terrestris* NRRL 8126 (XP_003653833.1), *Penicillium digitatum* Pd1 (EKV06812.1) and *Penicillium oxalicum* 114-2 (EPS30056.1).

## Abbreviations

CAZy: Carbohydrate Activity Enzyme database; DCW: dry cell weight; FGSC: Fungal Genetics Stock Center; HRC-1: hemicellulase regulator-1; MFS: major facilitator superfamily; NADH: nicotinamide adenine dinucleotide, reduced; NADPH: nicotinamide adenine dinucleotide phosphate, reduced; RPKM: reads per kilobase of exon model per million mapped reads; TCA cycle: tricarboxylic acid cycle; XR: D-xylose reductase; XDH: xylitol dehydrogenase; LAD: L-arabitol dehydrogenase; LXR: L-xylulose reductase; XKS: xylulokinase.

## Competing interests

The authors declare that they have no competing interests.

## Authors’ contributions

JL, CT and YM designed the project; JL, LL and HL carried out the experiments; JL, LL, HL, CT and YM wrote the manuscript. All authors read and approved the final manuscript.

## Supplementary Material

Additional file 1: Table S1 The profiles of RNA-seq reads mapped to the genome of *N. crassa* and differential expression analysis. Click here for file

Additional file 2: Figure S1 Correlation comparison between biological replicates under D-glucose **(A)** and L-arabinose **(B)** conditions. Normalized reads per kilobase of exon model per million mapped reads (RPKM) values used and spearman correlation coefficients (*r*) and *P*-values were calculated. Click here for file

Additional file 3: Table S2 Hierarchical clustering of genes with reads per kilobase of exon model per million mapped reads (RPKM) values >20 and showing significantly differential expression in *N. crassa* when grown on L-arabinose or D-xylose compared to that on D-glucose. Table S3 functional category analysis (FunCat) of genes grouped into cluster 1. Table S4 Functional category analysis (FunCat) of genes grouped into cluster 2. Table S5 functional category analysis (FunCat) of genes grouped into cluster 3. *P*-values highlighted in yellow indicate functional categories that were significantly enriched compared to the whole genome. Click here for file

Additional file 4: Table S6 The expression of Carbohydrate Activity Enzyme database (CAZy) genes significantly induced by L-arabinose. Click here for file

Additional file 5: Figure S2 Venn diagram of comparison of transcriptomes of *N. crassa* responsing to L-arabinose and no carbon source with that on D-glucose as the control. **(A)** Genes that showed increased expression under L-arabinose and/or no carbon conditions, compared to that on D-glucose. **(B)** Genes that showed decreased expression under L-arabinose and/or no carbon conditions. Click here for file

Additional file 6: Table S7 The genes showing up- or downregulated expression in *N.crassa* under L-arabinose and/or no carbon condition. Click here for file

Additional file 7: Figure S3 **(A)** Growth properties of recombinant *S. cerevisiae* EBY.VW4000 strains expressing different sugar transporter (GLT-1, LAT-1 and XYT-1) on D-glucose. Cells were spotted in serial dilutions on synthetic complete medium agar plates with 2% D-glucose. Cells transformed with the empty vector pRS426 served as a negative control. **(B)** Initial rates of sugar uptake of *S. cerevisiae* EBY.VW4000 expressing *glt-1* or *lat-1*. Pre-cultivated cells were incubated with radioactively labeled sugars (3 mM D-glucose, 3 mM D-xylose or 3 mM L-arabinose) for 2 min. The background values determined with cells containing the empty vector were subtracted. Click here for file

Additional file 8: Table S8 The expression levels of transcription factors showing significantly upregulated levels on L-arabinose. Table S9 Hemicellulase gene expression levels of the transcription factor deletion strains monitored by RT-PCR versus the wild-type strain when exposed to L-arabinose for 4 h. Click here for file

Additional file 9: Figure S4 Biomass production of wild-type (wt) and Δ*hcr-1* mutant. 10^8^ conidia from 10-day-old slants were collected and inoculated into 100 ml media (1 × Vogel’s salts with 2% carbon source (Avicel or xylan) in a 250-ml flask, then grown for 7 days at 25°C, with shaking at 200 rpm under constant light. Click here for file

Additional file 10: Figure S5 Neighbor-joining tree of HCR-1 homologs in filamentous fungi. The tree was generated by the MEGA5 program using neighbor joining with bootstrap = 1,000 (NCBI accession numbers of genes are given in the Methods). Click here for file
