# Supplementary figures and images for: Transcriptional comparison of the filamentous fungus Neurospora crassa growing on three major monosaccharides D-glucose, D-xylose and L-arabinose
Source: Biotechnol Biofuels. 2014 Feb 28;7:31. doi: 10.1186/1754-6834-7-31 (PMC4015282; doi:10.1186/1754-6834-7-31)

**A**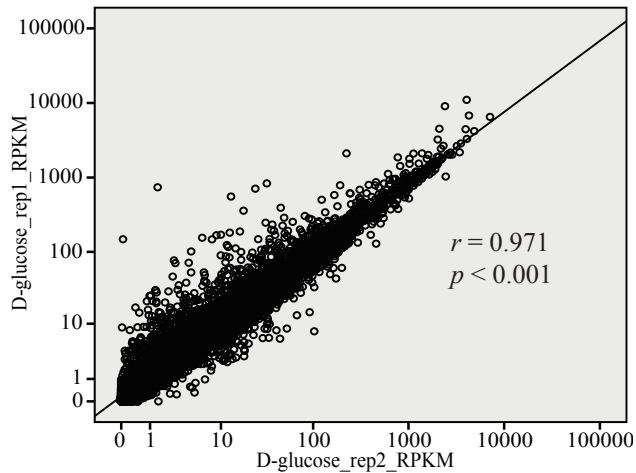**B**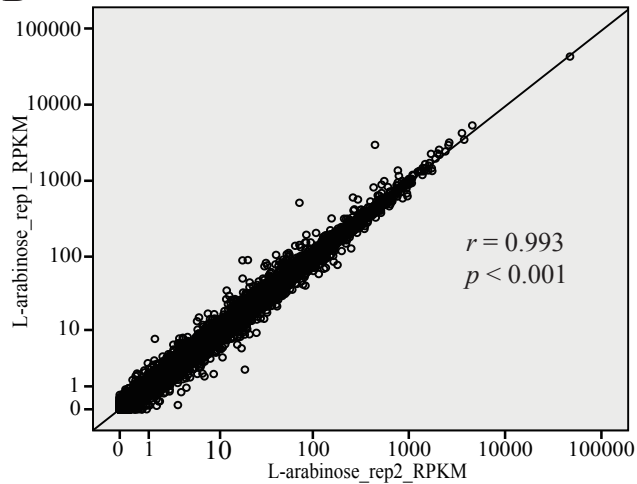

Supplement: Additional file 2: Figure S1 — Correlation comparison between biological replicates under D-glucose (A) and L-arabinose (B) conditions. Normalized reads per kilobase of exon model per million mapped reads (RPKM) values used and spearman correlation coefficients (r) and P-values were calculated. [file 1754-6834-7-31-S2.pdf]

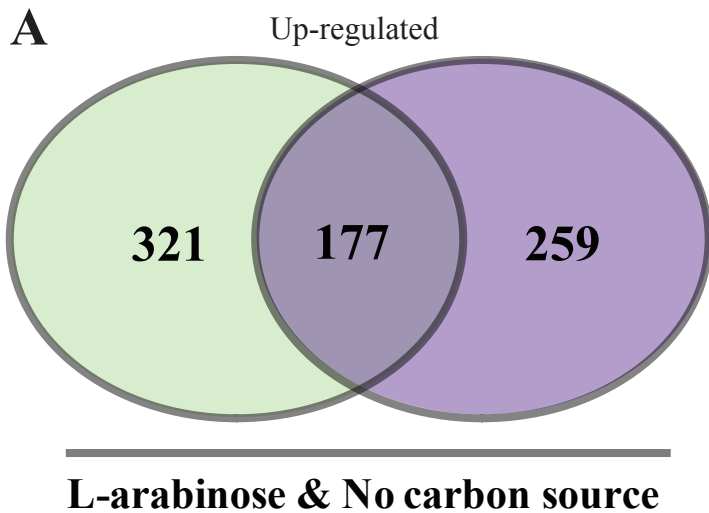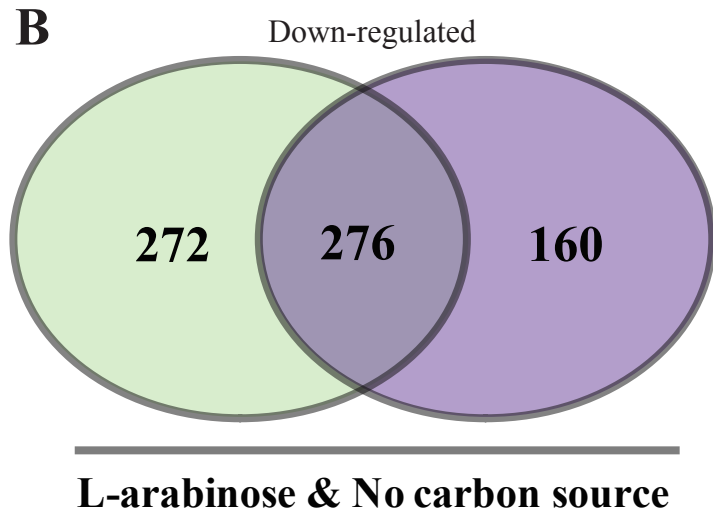

Supplement: Additional file 5: Figure S2 — Venn diagram of comparison of transcriptomes of N. crassa responsing to L-arabinose and no carbon source with that on D-glucose as the control. (A) Genes that showed increased expression under L-arabinose and/or no carbon conditions, compared to that on D-glucose. (B) Genes that showed decreased expression under L-arabinose and/or no carbon conditions. [file 1754-6834-7-31-S5.pdf]

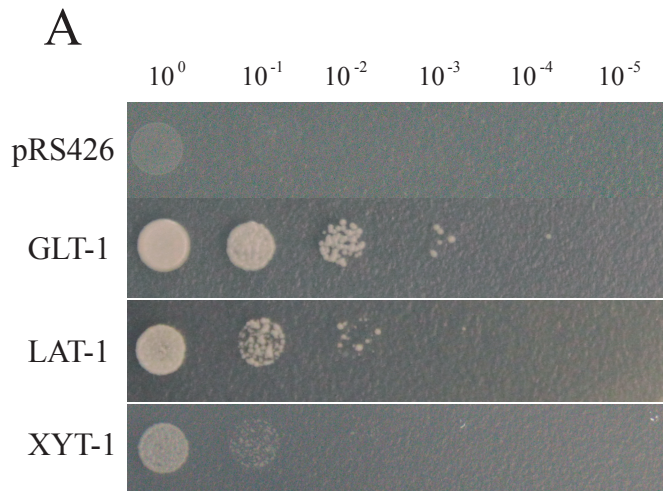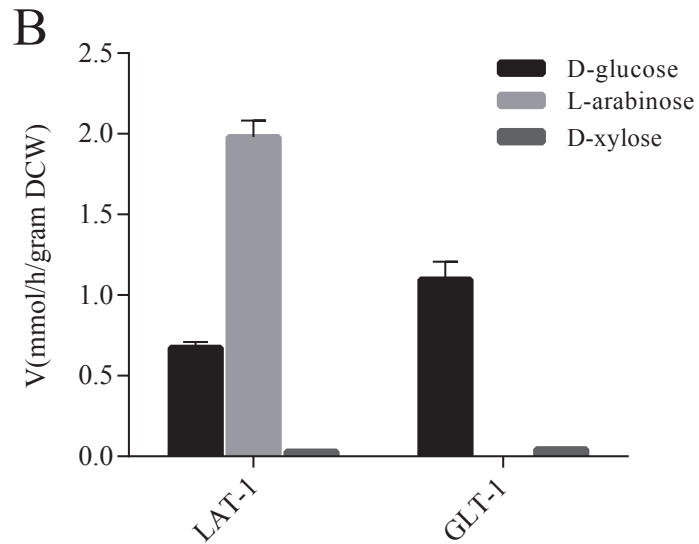

Supplement: Additional file 7: Figure S3 — (A) Growth properties of recombinant S. cerevisiae EBY.VW4000 strains expressing different sugar transporter (GLT-1, LAT-1 and XYT-1) on D-glucose. Cells were spotted in serial dilutions on synthetic complete medium agar plates with 2% D-glucose. Cells transformed with the empty vector pRS426 served as a negative control. (B) Initial rates of sugar uptake of S. cerevisiae EBY.VW4000 expressing glt-1 or lat-1. Pre-cultivated cells were incubated with radioactively labeled sugars (3 mM D-glucose, 3 mM D-xylose or 3 mM L-arabinose) for 2 min. The background values determined with cells containing the empty vector were subtracted. [file 1754-6834-7-31-S7.pdf]

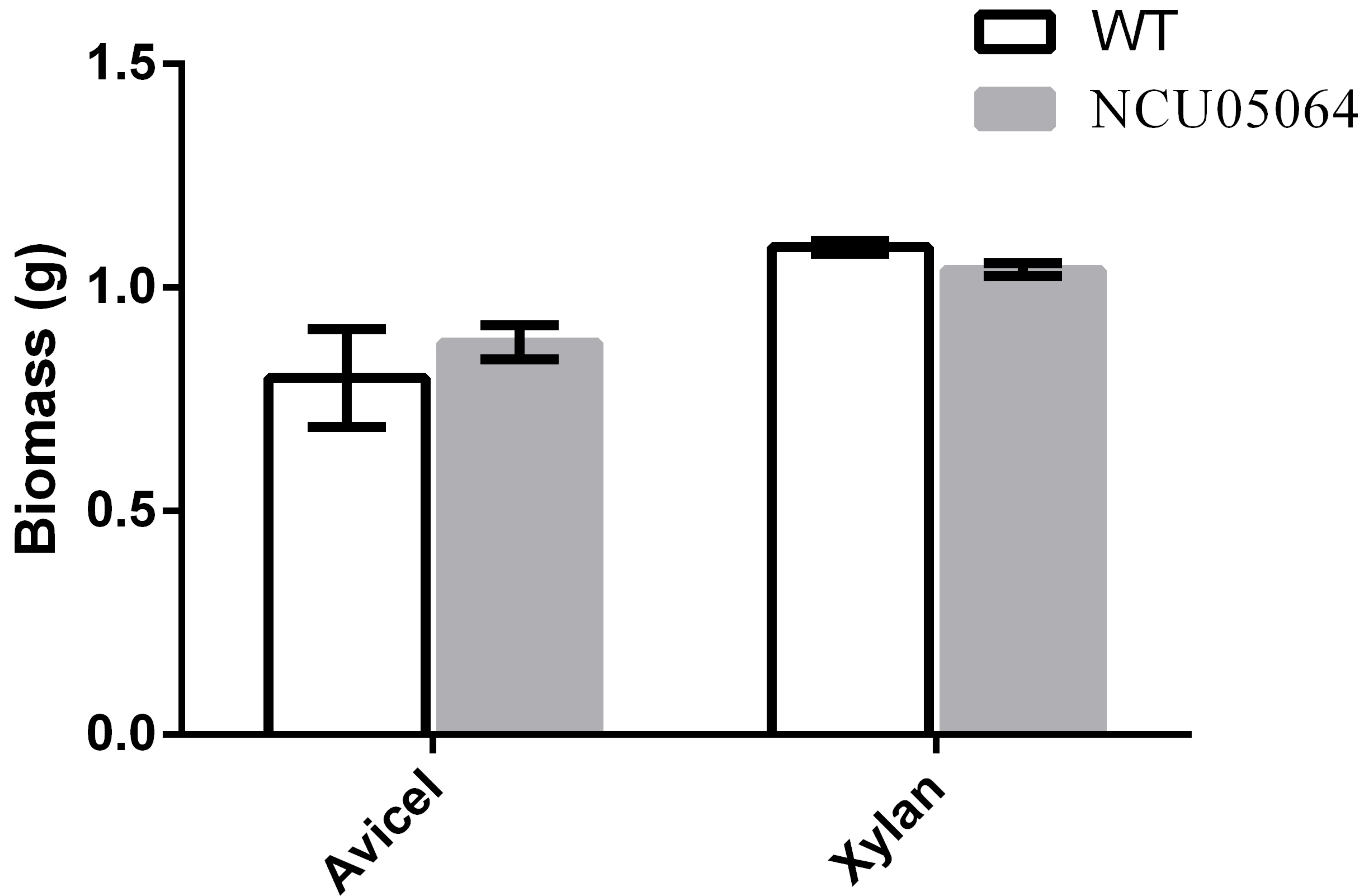

Supplement: Additional file 9: Figure S4 — Biomass production of wild-type (wt) and Δhcr-1 mutant. 108 conidia from 10-day-old slants were collected and inoculated into 100 ml media (1 × Vogel’s salts with 2% carbon source (Avicel or xylan) in a 250-ml flask, then grown for 7 days at 25°C, with shaking at 200 rpm under constant light. [file 1754-6834-7-31-S9.pdf]

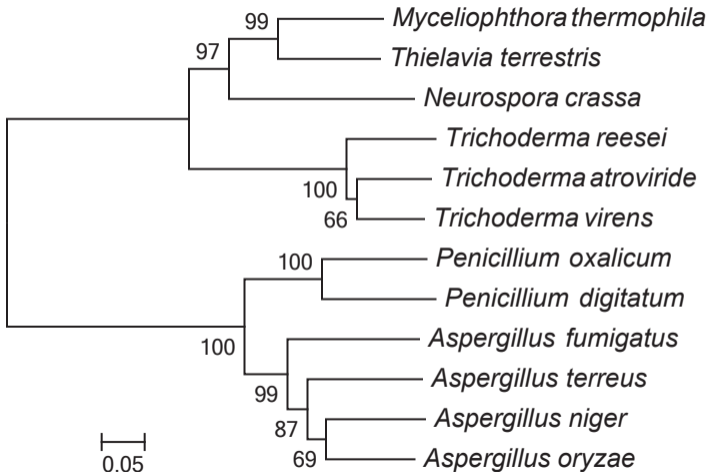

Supplement: Additional file 10: Figure S5 — Neighbor-joining tree of HCR-1 homologs in filamentous fungi. The tree was generated by the MEGA5 program using neighbor joining with bootstrap = 1,000 (NCBI accession numbers of genes are given in the Methods). [file 1754-6834-7-31-S10.pdf]
